# Seroprevalence of Hepatitis A Virus and Hepatitis E Virus Among Patients With Acute Viral Hepatitis in a Tertiary Care Hospital: A Retrospective Study

**DOI:** 10.7759/cureus.107739

**Published:** 2026-04-26

**Authors:** Kunalsen Jagatdeo, Ankita Roy, Kumudini Panigrahi, Nishikanta Thatoi, Jyoti Prakash Sahoo, Basanti Kumari Pathi

**Affiliations:** 1 Microbiology, Kalinga Institute of Medical Sciences, Bhubaneswar, IND; 2 Pharmacology, Kalinga Institute of Medical Sciences, Bhubaneswar, IND

**Keywords:** acute viral hepatitis, fecal-oral transmitted hepatitis, fever with jaundice, hepatitis a virus (hav), hepatitis e virus (hev), igm antibodies, liver enzyme levels, seroprevalence, serum total bilirubin, trend analysis

## Abstract

Background and objectives: Hepatitis A virus (HAV) and hepatitis E virus (HEV) infections cause acute viral hepatitis. Increased cases of HAV and HEV infection in the Indian subcontinent could be attributed to the region's large population. We conducted this study to determine the seroprevalence of HAV and HEV infections in our hospital. We also evaluated the symptoms (e.g., fever, jaundice, pain abdomen, and loose motion), liver enzymes like serum glutamic-oxaloacetic transaminase (SGOT) and serum glutamic-pyruvic transaminase (SGPT), and serum total bilirubin levels of the study participants.

Methods: This retrospective study analyzed the patients with suspected acute viral hepatitis who visited Kalinga Institute of Medical Sciences (KIMS), Bhubaneswar, India, between January 2023 and November 2025. We included the blood samples received for anti-HAV IgM and anti-HEV IgM testing during the study period. We presented the symptoms and their durations through highlight plots. The trend of symptoms was assessed for the three years. We demonstrated SGOT, SGPT, and serum total bilirubin levels in patients with HAV-, HEV-, and co-infection through violin and box-and-whisker plots. Their trends were also analyzed for the three years.

Results: We analyzed 3,607 samples from patients suspected of acute viral hepatitis. The median age was 25.0 (interquartile range (IQR), 16.0-43.0) years. The study population had 1346 (37.32%) female and 2261 (62.68%) male patients. In 2023, 2024, and 2025, there were 878, 1189, and 1540 acute viral hepatitis cases, respectively. Abdominal pain was the most common presenting symptom (n=1703, 47.21%), followed by fever (n=1529, 42.38%), jaundice (n=1375, 38.12%), and loose motion (n=441, 12.22%). HAV and HEV infections were seen in 1567 (43.44%) and 147 (4.08%) participants, respectively. A total of 134 (3.71%) participants had both infections, and the remaining 1759 (48.77%) were negative for both viruses. The median SGOT values of participants with HAV, HEV, and both infections were 508.40 (IQR, 406.70-930.75) IU/L, 502.70 (IQR, 409.40-1631.70) IU/L, and 516.95 (IQR, 416.90-1155.70) IU/L, respectively. The median SGPT values of participants with HAV, HEV, and both infections were 543.90 (IQR, 404.45-898.05) IU/L, 532.20 (IQR, 392.70-1722.35) IU/L, and 544.70 (IQR, 393.43-1077.30) IU/L, respectively. The median serum total bilirubin values of participants with HAV, HEV, and both infections were 5.07 (IQR, 2.92-7.22) mg/dL, 5.42 (IQR, 3.11-7.31) mg/dL, and 4.72 (IQR, 2.92-6.87) mg/dL, respectively.

Conclusion: The number of cases increased with each year. HAV was more common than HEV and co-infection. The liver enzymes and serum total bilirubin levels were elevated in infected participants compared with non-infected participants. Owing to India's large population, HAV and HEV infections are serious public health concerns. Early detection, diagnosis, and treatment of suspected cases of HAV and HEV infection could thereby lessen the strain on hospitals and lower morbidity and death.

## Introduction

Hepatitis A virus (HAV) and Hepatitis E virus (HEV) are significant contributors to acute viral hepatitis in developing nations such as India [1,2]. Viral hepatitis often causes lethargy, anorexia, jaundice, abdominal pain, loose motion, and fever [1,3,4]. There are two types of hepatitis infection: acute and chronic. Chronic hepatitis lasts longer than six months, while acute hepatitis does not [3]. The transmission of HAV and HEV is facilitated by inadequate sanitation, poor hygiene, and contaminated drinking water via the fecal-oral route [1,3].

HAV is a non-enveloped, single-stranded RNA virus belonging to the *Picornaviridae* family and the genus *Hepatovirus* [2,4,5]. HEV is a non-enveloped, positive-sense single-stranded RNA virus that belongs to the *Hepeviridae* family [6]. HAV and HEV infections are major health issues among rural residents of India [2,7].

Serological diagnosis of acute HAV and HEV infections primarily relies on the detection of anti-HAV IgM and anti-HEV IgM antibodies, respectively [2,8]. These antibodies appear early in the course of illness and remain detectable for several months [9]. A recent study by Mahadevaiah et al. found that the incidences of HAV, HEV, and co-infection were 62.9%, 25.8%, and 11.2%, respectively [10]. A recent meta-analysis of 28 studies from 2000 to 2021 by Kumar et al. found that the incidences of HAV and HEV ranged 2.1-52.5% and 10.54-68.42%, respectively [11]. They also found that HAV and HEV infections were more common in children and third-trimester pregnant women, respectively. Acute HAV and HEV infections cause increased liver enzymes, such as serum glutamic-oxaloacetic transaminase (SGOT) and serum glutamic-pyruvic transaminase (SGPT), and increased serum total bilirubin levels [12-14].

Many studies have reported notable regional, demographic, and seasonal differences in the prevalence of acute viral hepatitis. This indicates that epidemiological research should be conducted on specific populations [2,5,15-17]. In this context, the present study was undertaken to estimate the seroprevalence of HAV and HEV among clinically suspected cases of acute viral hepatitis attending a tertiary care hospital in eastern India over the last three years. We aimed to determine symptom patterns for HAV, HEV, and co-infection. We also measured SGOT, SGPT, and serum total bilirubin in the participants.

## Materials and methods

In this retrospective study, we analyzed the patients with suspected acute viral hepatitis who visited Kalinga Institute of Medical Sciences (KIMS), Bhubaneswar, Odisha, India, between January 2023 and November 2025. The Institutional Ethics Committee, KIMS, granted ethical clearance to begin the study (reference number: KIIT/KIMS/IEC/2382/2025, dated December 1, 2025). Informed consent was not required owing to the retrospective nature of the study.

Eligibility criteria

We checked the laboratory data of all patients from the outpatient department and inpatient wards whose blood samples were sent to the microbiology laboratory for serological evaluation of suspected acute viral hepatitis. Of them, the samples received for anti-HAV IgM and anti-HEV IgM testing during the study period were included in the analysis. Incomplete records were excluded. One sample per patient was analyzed in this study.

Study procedure

Demographic, clinical, and laboratory data of the participants were retrieved retrospectively from hospital records and laboratory registers. Demographic data included age, gender, and place of residence (i.e., rural or urban). The clinical data included the patients' symptoms (i.e., fever, loose motion, jaundice, and abdominal pain). The laboratory data included serum transaminase values (i.e., SGOT and SGPT), serum total bilirubin, anti-HAV IgM, and anti-HEV IgM status of the patients. These data were compiled into a structured electronic database for analysis.

As part of routine clinical practices, blood samples from clinically suspected cases were processed in the microbiology laboratory for serological detection of acute HAV and HEV infection. After the clot formation, the blood samples were centrifuged, and serum was separated using standard laboratory procedures. The separated serum was subjected to an enzyme-linked immunosorbent assay (ELISA) test for the detection of anti-HAV IgM and anti-HEV IgM antibodies [2,8]. Commercially available ELISA kits (Dia.Pro HAV IgM and HEV IgM ELISA kits; Dia.Pro Diagnostic Bioprobes SRL, Sesto San Giovanni, Italy) were used to detect these antibodies.

The assessments through these ELISA kits were based on the IgM capture principle [18,19]. Serum samples (1:101 dilution) were incubated in anti-IgM-coated wells, followed by the addition of enzyme-conjugated antigen (inactivated hepatitis A or E antigen) and substrate. The optical density (OD) value of the sample was read at 450 nm. The cut-off value was computed as the OD value for the negative control + 2.5. IgM levels were expressed as the ratio of the sample OD to the cut-off value [20]. Anti-HAV IgM values < 0.8, 0.8-1.2, and >1.2 were interpreted as negative, equivocal, and positive, respectively. Similarly, anti-HEV IgM values < 1.0, 1.0-1.2, and > 1.2 were interpreted as negative, equivocal, and positive, respectively [20]. The samples with “equivocal” status were retested with a second sample after one to two weeks from the initial test. The samples with either positive or negative results were included in the analysis. The equivocal results were excluded. The sensitivity and specificity of the test kit are 95-100% and 98-100%, respectively. SGOT levels less than 35 IU/L are considered normal. The typical range for SGPT is 19-25 IU/L for females and 29-33 IU/L for males [13].

Statistical analysis

This retrospective study employed convenience sampling. The Kolmogorov-Smirnov test assessed the normality of the data distribution. The continuous data were expressed as median and interquartile range (IQR). The categorical data were expressed as frequency and proportion. We analyzed the categorical data with the chi-square test and presented the chi-square values. The Kruskal-Wallis test was used to analyze the continuous data, and H-values were reported. The post hoc analysis was performed through Bonferroni correction. We generated highlight plots to assess the symptoms. The serum transaminase values (i.e., SGOT and SGPT) and serum total bilirubin were illustrated using violin and box-and-whisker plots. The R program version 4.5.3 (R Foundation for Statistical Computing, Vienna, Austria) was used for data analysis and plot generation [21]. P-values less than 0.05 were interpreted as statistically significant.

## Results

We analyzed data of 3607 patients with clinically suspected acute viral hepatitis. Table 1 shows the demographic traits and presenting symptoms of the study population. In the years 2023, 2024, and 2025, the number of patients with acute viral hepatitis visiting KIMS was 878, 1189, and 1540, respectively. There was an increasing trend in the number of cases over time. The median age of the study population was 25 (IQR, 16-43) years. In 2025, the median age dropped to 22 (IQR, 12-41) years. There was a male preponderance (n=2261, 62.68%). Abdominal pain was the most common presenting symptom (n=1703, 47.21%), followed by fever (n=1529, 42.38%), jaundice (n=1375, 38.12%), and loose motion (n=441, 12.22%).

**Table 1:** Demographic and clinical details of the study population The continuous data were presented as medians and IQRs. The categorical data were presented as frequency and percentage. The continuous data were assessed using the Kruskal-Wallis test, and H-values were reported. Categorical data were assessed using the Chi-square test, and the Chi-square values were reported. Statistical significance was set at p < 0.05. IQR: interquartile range

Variable	Total (n = 3607)	2023 (n = 878)	2024 (n = 1189)	2025 (n = 1540)	Statistical test used	Test statistics	p-value
Age (years), mean (IQR)	25 (16-43)	26 (19-45)	28 (19-44)	22 (12-41)	Kruskal-Wallis test	23.952	< 0.001
Gender, n (%)							
Male	2261 (62.68%)	572 (65.14%)	776 (65.26%)	913 (59.28%)	Chi-square test	16.634	< 0.001
Female	1346 (37.32%)	306 (34.86%)	413 (34.74%)	627 (40.72%)			
Residence, n (%)							
Rural	2478 (68.70%)	625 (71.18%)	878 (73.84%)	975 (63.31%)	Chi-square test	46.833	< 0.001
Urban	1129 (31.30%)	253 (28.82%)	311 (26.16%)	565 (36.69%)			
Past history of hepatitis	561 (15.55%)	186 (21.18%)	166 (13.96%)	209 (13.57%)	Chi-square test	21.704	< 0.001
Presenting symptoms, n (%)							
Fever	1529 (42.38%)	383 (43.62%)	508 (42.72%)	638 (41.42%)	Chi-square test	68.097	< 0.001
Loose motion	441 (12.22%)	104 (11.84%)	144 (12.11%)	193 (12.53%)			
Jaundice	1375 (38.12%)	335 (38.15%)	448 (37.67%)	592 (38.44%)			
Abdominal pain	1703 (47.21%)	420 (47.84%)	565 (47.51%)	718 (46.62%)			

Trend analysis of symptoms

**Figure 1:**
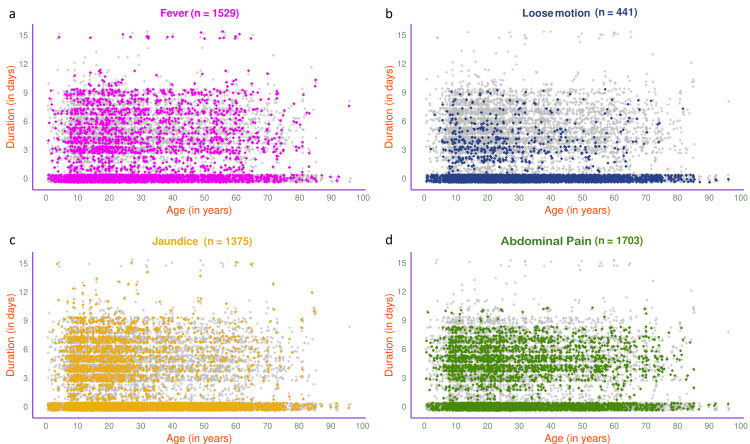
Symptoms of viral hepatitis among all study participants (n = 3607) The jitter plots illustrate the patterns of (a) fever, (b) loose motion, (c) jaundice, and (d) abdominal pain for all participants (n = 3607) in different colors. The x- and y-axes denote participants’ age (in years) and symptom duration (in days), respectively.

**Figure 2:**
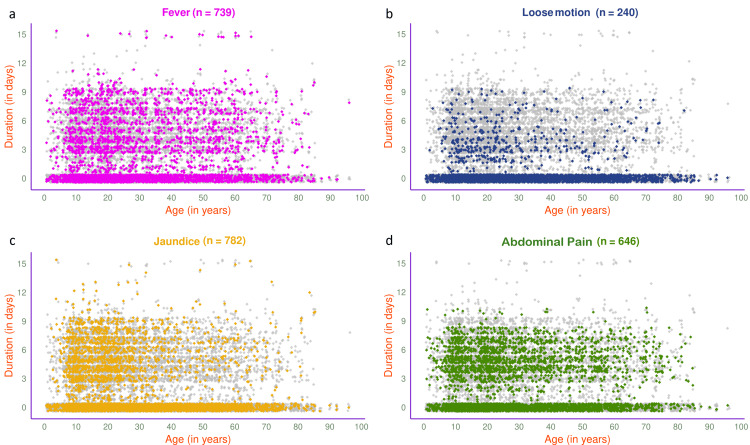
Patterns of symptoms among HAV- and/or HEV-positive participants (n = 1848) The jitter plots illustrate the patterns of (a) fever, (b) loose motion, (c) jaundice, and (d) abdominal pain in HAV- and/or HEV-positive participants (n = 1848) in different colors.. The x- and y-axes denote participants’ age (in years) and symptom duration (in days), respectively.

The jitter plots in Figure 1 illustrate the symptoms of 3607 participants and their durations. The symptoms (i.e., fever, loose motion, jaundice, and abdominal pain) are shown in different colours. The most common presenting symptom was abdominal pain (n=1703, 47.21%), followed by fever (n=1529, 42.38%), jaundice (n=1375, 38.12%), and loose motion (n=441, 12.22%). The jitter plots in Figure 2 illustrate the symptoms of 1848 participants with HAV, HEV, or both. Jaundice (n=782, 42.32%) was the most common presenting symptom, followed by fever (n=739, 39.99%), abdominal pain (n=646, 34.96%), and loose motion (n=240, 12.99%).

**Figure 3:**
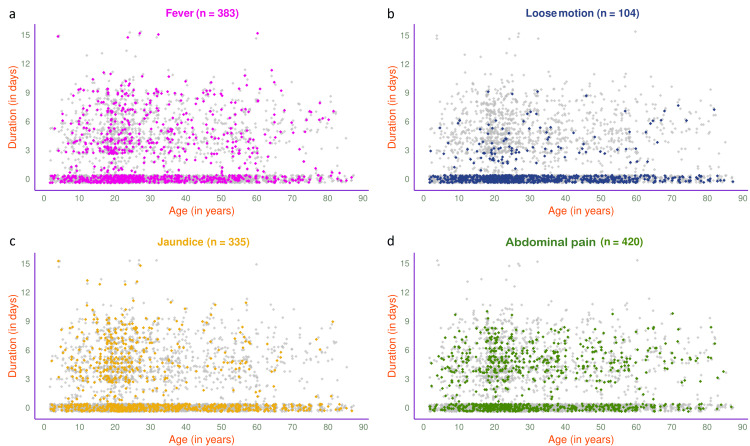
Symptoms of viral hepatitis among the study participants in 2023 (n = 878) The jitter plots illustrate the patterns of (a) fever, (b) loose motion, (c) jaundice, and (d) abdominal pain for all participants in 2023 (n = 878) in different colours. The x- and y-axes denote participants’ age (in years) and symptom duration (in days), respectively.

**Figure 4:**
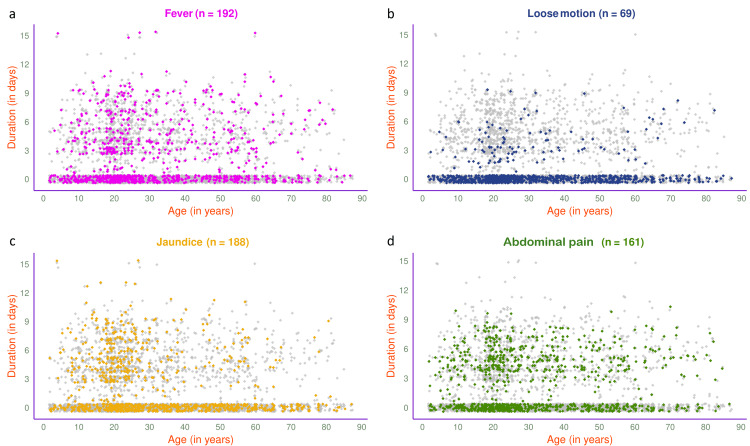
Symptoms of viral hepatitis among HAV- and/or HEV-positive participants in 2023 (n = 452) The jitter plots illustrate the patterns of (a) fever, (b) loose motion, (c) jaundice, and (d) abdominal pain among HAV- and/or HEV-positive participants in 2023 (n = 452) in different colours. The x- and y-axes denote participants’ age (in years) and symptom duration (in days), respectively.

The jitter plots in Figure 3 illustrate the symptoms of 878 participants in 2023. The most common presenting symptom was abdominal pain (n=420, 47.84%), followed by fever (n=383, 43.62%), jaundice (n=335, 38.15%), and loose motion (n=104, 11.85%). The jitter plots in Figure 4 illustrate the symptoms of 452 participants in 2023 with HAV, HEV, or both. Fever (n=192, 42.48%) was the most common presenting symptom, followed by jaundice (n=188, 41.59%), abdominal pain (n=161, 35.62%), and loose motion (n=69, 15.27%).

**Figure 5:**
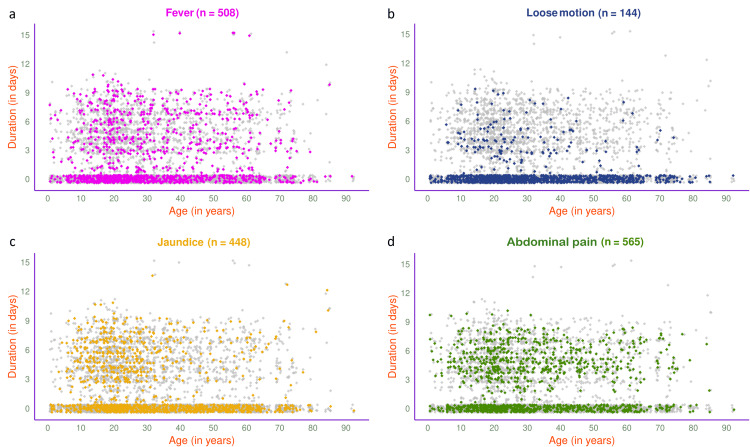
Symptoms of viral hepatitis among the study participants in 2024 (n = 1189) The jitter plots illustrate the pattern of (a) fever, (b) loose motion, (c) jaundice, and (d) abdominal pain for all participants in 2024 (n = 1189) in different colours. The x- and y-axes denote participants’ age (in years) and symptom duration (in days), respectively.

**Figure 6:**
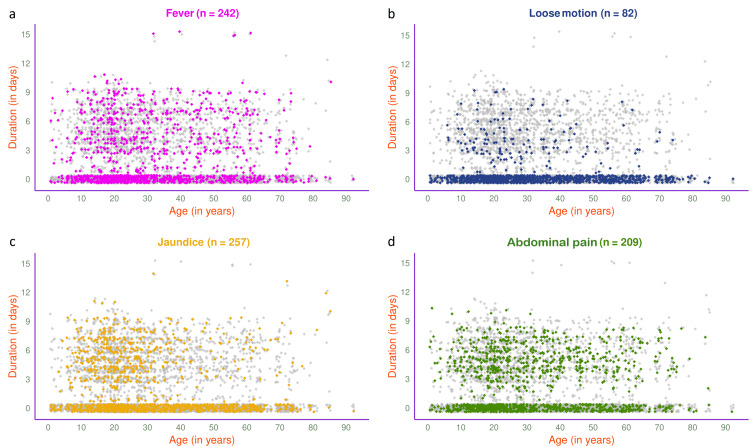
Symptoms of viral hepatitis among HAV- and/or HEV-positive participants in 2024 (n = 593) The jitter plots illustrate the patterns of (a) fever, (b) loose motion, (c) jaundice, and (d) abdominal pain for HAV- and/or HEV-positive participants in 2024 (n = 593) in different colours. The x- and y-axes denote participants’ age (in years) and symptom duration (in days), respectively.

The jitter plots in Figure 5 illustrate the symptoms of 1189 participants in 2024. The most common presenting symptom was abdominal pain (n=565, 47.52%), followed by fever (n=508, 42.72%), jaundice (n=448, 37.68%), and loose motion (n=144, 12.11%). The jitter plots in Figure 6 illustrate the symptoms of 593 participants in 2024 with HAV, HEV, or both. Jaundice (n=257, 43.34%) was the most common presenting symptom, followed by fever (n=242, 40.81%), abdominal pain (n=209, 35.24%), and loose motion (n=82, 13.83%).

**Figure 7:**
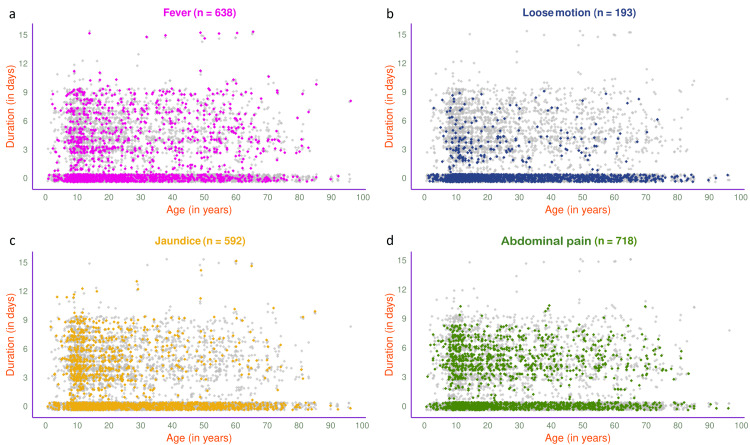
Symptoms of viral hepatitis among the study participants in 2025 (n = 1540) The jitter plots illustrate the patterns of (a) fever, (b) loose motion, (c) jaundice, and (d) abdominal pain for all participants in 2025 (n = 1540) in different colours. The x- and y-axes denote participants’ age (in years) and symptom duration (in days), respectively.

**Figure 8:**
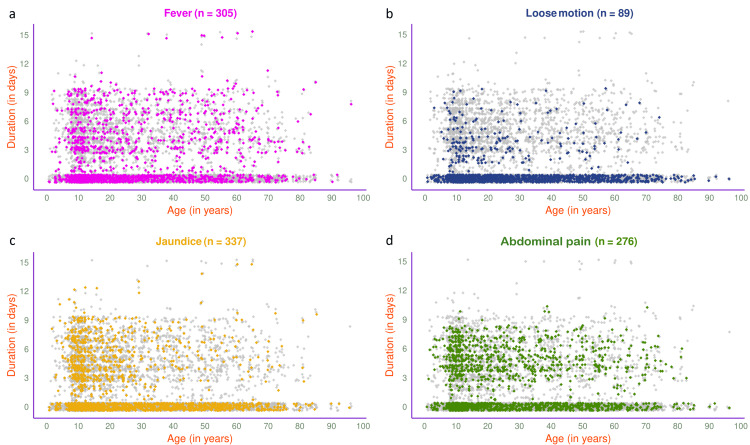
Symptoms of viral hepatitis among HAV- and/or HEV-positive participants in 2025 (n = 803) The jitter plots illustrate the patterns of (a) fever, (b) loose motion, (c) jaundice, and (d) abdominal pain for HAV- and/or HEV-positive participants in 2025 (n = 803) in different colours. The x- and y-axes denote participants’ age (in years) and symptom duration (in days), respectively.

The jitter plots in Figure 7 illustrate the symptoms of 1540 participants in 2025. The most common presenting symptom was abdominal pain (n=718, 46.62%), followed by fever (n=638, 41.43%), jaundice (n=592, 38.44%), and loose motion (n=193, 12.53%). The jitter plots in Figure 8 illustrate the symptoms of 803 participants in 2025 with HAV, HEV, or both. Jaundice (n=337, 41.97%) was the most common presenting symptom, followed by fever (n=305, 37.98%), abdominal pain (n=276, 34.37%), and loose motion (n=89, 11.08%).

Analysis of symptoms among participants with HAV, HEV, or both

Table 2 shows the symptom distribution of patients positive with HAV. Among the 1567 HAV-positive cases, 372 were recorded in 2023, 503 in 2024, and 692 in 2025. Jaundice was the most common symptom, present in 656 cases (41.86%), followed by fever in 614 (39.18%), abdominal pain in 533 (34.01%), and loose motion in 198 (12.63%).

**Table 2:** Symptom distribution among HAV-positive cases Data are presented as frequency and percentage, and were assessed using the chi-square test; chi-square values are reported. Statistical significance was set at p < 0.05. HAV: hepatitis A virus

Symptoms	Total (n = 1567)	2023 (n = 372)	2024 (n = 503)	2025 (n = 692)	Chi-square value	p-value
Fever	614 (39.18%)	154 (41.39%)	199 (39.56%)	261 (37.71%)	36.852	< 0.001
Loose motion	198 (12.63%)	55 (14.78%)	67 (13.32%)	76 (10.98%)	24.039	< 0.001
Jaundice	656 (41.86%)	155 (41.66%)	215 (42.74%)	286 (41.32%)	51.703	< 0.001
Pain abdomen	533 (34.01%)	127 (34.13%)	174 (34.59%)	232 (33.52%)	47.496	< 0.001

Table 3 shows the symptom distribution of patients positive with HEV. Among the 147 HEV-positive cases, 46 occurred in 2023, 49 in 2024, and 52 in 2025. Fever was the most common presenting symptom, seen in 73 cases (49.65%), followed by jaundice in 71 (48.29%), abdominal pain in 70 (47.61%), and loose motion in 27 (18.36%).

**Table 3:** Symptom distribution among HEV-positive cases Data are presented as frequency and percentage, and were assessed using the chi-square test; chi-square values are reported. Statistical significance was set at p < 0.05. HEV: hepatitis E virus

Symptoms	Total (n = 147)	2023 (n = 46)	2024 (n = 49)	2025 (n = 52)	Chi-square value	p-value
Fever	73 (49.65%)	21 (45.65%)	26 (53.06%)	26 (50.00%)	11.054	< 0.001
Loose motion	27 (18.36%)	11 (23.91%)	8 (16.32%)	8 (15.38%)	10.402	< 0.001
Jaundice	71 (48.29%)	20 (43.47%)	25 (51.02%)	26 (50.00%)	13.724	< 0.001
Pain abdomen	70 (47.61%)	21 (45.65%)	26 (53.06%)	23 (44.23%)	11.391	< 0.001

Table 4 shows the symptom distribution of patients who are positive for both HAV and HEV. Among the 134 HAV-HEV co-infected cases, 34 were identified in 2023, 41 in 2024, and 59 in 2025. Jaundice was the most frequent symptom, occurring in 55 cases (41.04%), followed by fever in 52 (38.80%), abdominal pain in 43 (32.08%), and loose motion in 15 (11.19%).

**Table 4:** Symptom distribution among both HAV- and HEV-positive cases Data are presented as frequency and percentage, and were assessed using the chi-square test; chi-square values are reported. Statistical significance was set at p < 0.05. HAV: hepatitis A virus; HEV: hepatitis E virus

Symptoms	Total (n = 134)	2023 (n = 34)	2024 (n = 41)	2025 (n = 59)	Chi-square value	p-value
Fever	52 (38.80%)	17 (50.00%)	17 (41.46%)	18 (30.50%)	5.806	< 0.001
Loose motion	15 (11.19%)	3 (8.82%)	7 (17.07%)	5 (8.47%)	6.219	< 0.001
Jaundice	55 (41.04%)	13 (38.23%)	17 (41.46%)	25 (42.37%)	12.877	< 0.001
Pain abdomen	43 (32.08%)	13 (38.23%)	9 (21.95%)	21 (35.59%)	28.843	< 0.001

Analysis of serum transaminases and total bilirubin

**Figure 9:**
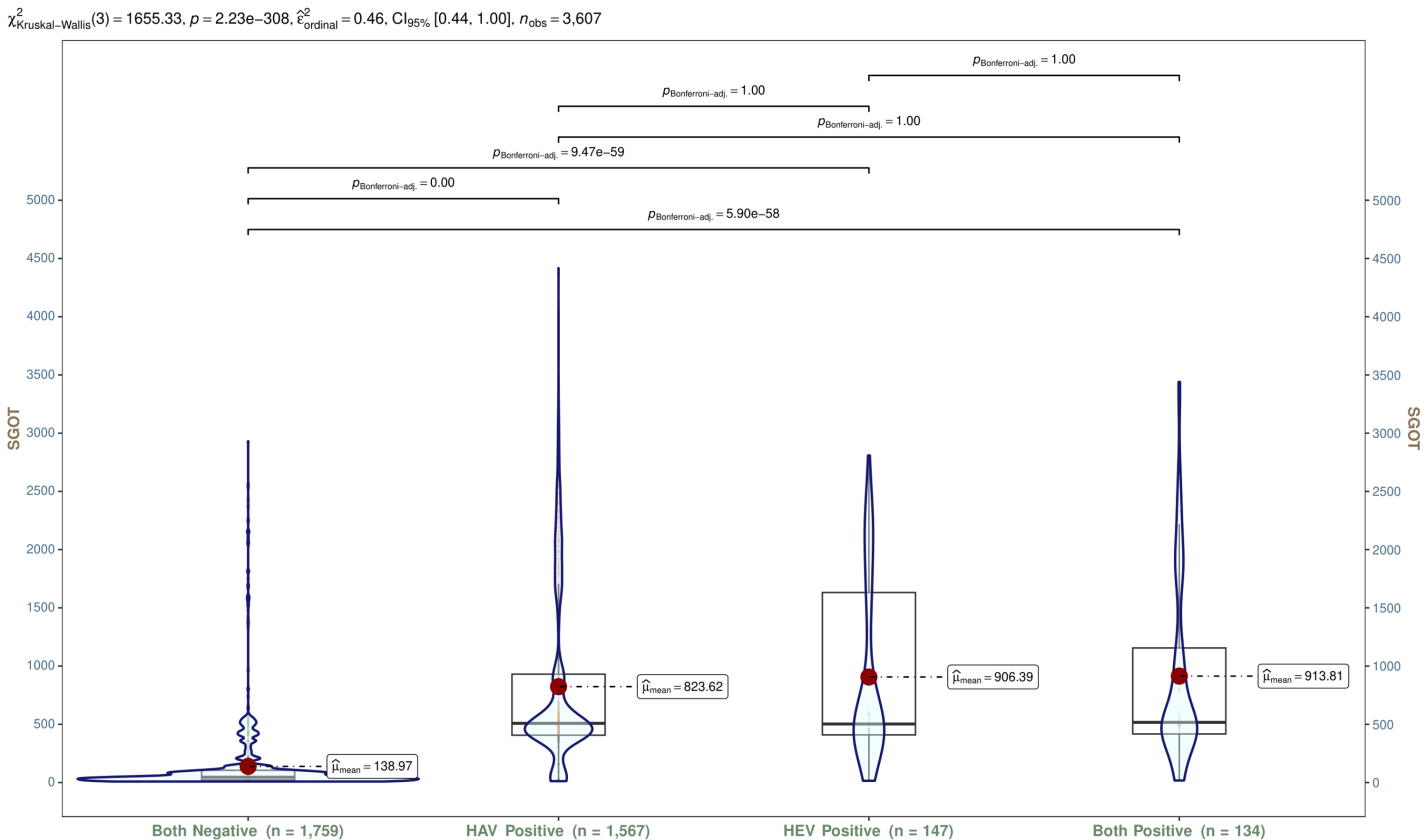
SGOT values of all participants The box-and-whisker and violin plots illustrate SGOT values (IU/L) of all participants (n = 3607). The normal range of SGOT is < 35 IU/L. The mean values are shown as red dots. The Kruskal-Wallis test was used to analyze these variables, and post hoc analysis was done with the Bonferroni adjustment. Statistical significance was set at p < 0.05. HAV: hepatitis A virus; HEV: hepatitis E virus; SGOT: serum glutamic-oxaloacetic transaminase

In Figure 9, SGOT values of all participants are shown through violin and box-and-whisker plots. The median SGOT values of participants with HAV, HEV, and both infections were 508.40 (IQR, 406.70-930.75) IU/L, 502.70 (IQR, 409.40-1631.70) IU/L, and 516.95 (IQR, 416.90-1155.70) IU/L, respectively. The Kruskal-Wallis test and post hoc analysis with the Bonferroni adjustment revealed statistically significant differences (p < 0.001). Tables 5-7 show SGOT values for participants with HAV, HEV, and both infections, respectively. The trend analyses of these participants showed statistically significant differences (p < 0.001).

**Table 5:** Liver enzymes and bilirubin levels of HAV-positive cases The continuous variables were reported as medians and IQRs. The Kruskal-Wallis test was used to analyze these variables, and H-values were mentioned. Statistical significance was set at p < 0.05. HAV: hepatitis A virus; SGOT: serum glutamic-oxaloacetic transaminase; SGPT: serum glutamic-pyruvic transaminase; IQR: interquartile range

Parameters	Total (n = 1567)	2023 (n = 372)	2024 (n = 503)	2025 (n = 692)	H-value	p-value
SGOT (IU/L)	508.40 (406.70-930.75)	484.70 (399.28-981.13)	512.40 (415.45-895.40)	517.25 (409.68-923.70)	28.937	< 0.001
SGPT (IU/L)	543.90 (404.45-898.05)	531.70 (379.50-926.50)	525.40 (405.50-885.00)	553.85 (413.65-889.60)	12.497	< 0.001
Total bilirubin (mg/dL)	5.07 (2.92-7.22)	5.41 (2.84-7.89)	5.42 (3.06-7.66)	4.79 (1.74-6.87)	9.063	< 0.001

**Table 6:** Liver enzymes and bilirubin levels of HEV-positive cases The continuous variables were reported as medians and IQRs. The Kruskal-Wallis test was used to analyze these variables, and H-values were mentioned. Statistical significance was set at p < 0.05. HEV: hepatitis E virus; SGOT: serum glutamic-oxaloacetic transaminase; SGPT: serum glutamic-pyruvic transaminase; IQR: interquartile range

Parameters	Total (n = 147)	2023 (n = 46)	2024 (n = 49)	2025 (n = 52)	H-value	p-value
SGOT (IU/L)	502.70 (409.40-1631.70)	499.00 (381.30-1357.5)	498.60 (418.50-902.50)	552.00 (408.00-1659.23)	14.078	< 0.001
SGPT (IU/L)	532.20 (392.70-1722.35)	517.35 (425.93-1265.80)	520.20 (384.00-924.20)	566.55 (374.78-1828.90)	23.714	< 0.001
Total bilirubin (mg/dL)	5.42 (3.11-7.31)	4.90 (3.15-10.28)	5.42 (3.10-6.79)	5.57 (3.25-6.98)	7.848	< 0.001

**Table 7:** Liver enzymes and bilirubin levels of both HAV- and HEV-positive cases The continuous variables were reported as medians and IQRs. The Kruskal-Wallis test was used to analyze these variables, and H-values were mentioned. Statistical significance was set at p < 0.05. HAV: hepatitis A virus; HEV: hepatitis E virus; SGOT: serum glutamic-oxaloacetic transaminase; SGPT: serum glutamic-pyruvic transaminase; IQR: interquartile range

Parameters	Total (n = 134)	2023 (n = 34)	2024 (n = 41)	2025 (n = 59)	H-value	p-value
SGOT (IU/L)	516.95 (416.90-1155.70)	491.95 (416.58-926.48)	870.00 (487.50-1531.20)	501.00 (413.75-1063.35)	67.814	< 0.001
SGPT (IU/L)	544.70 (393.43-1077.30)	499.85 (359.05-862.25)	848.30 (468.70-1349.50)	489.90 (347.65-984.95)	63.219	< 0.001
Total bilirubin (mg/dL)	4.72 (2.92-6.87)	4.90 (1.55-6.87)	4.72 (3.30-6.87)	4.72 (2.99-6.87)	3.043	< 0.001

**Figure 10:**
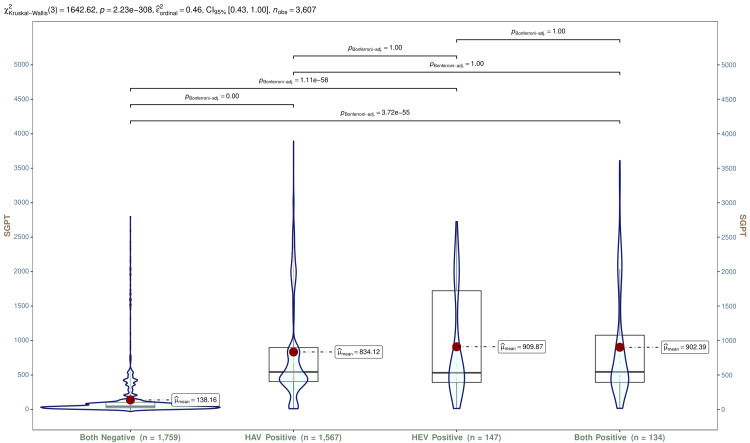
SGPT values of all participants The box-and-whisker and violin plots illustrate SGPT values (IU/L) of all participants (n = 3607). The normal range of SGPT is 19–25 IU/L for females and 29–33 IU/L for males. The mean values are shown as red dots. The Kruskal-Wallis test was used to analyze these variables, and post hoc analysis was done with the Bonferroni adjustment. Statistical significance was set at p < 0.05. HAV: hepatitis A virus; HEV: hepatitis E virus; SGPT: serum glutamic-pyruvic transaminase

In Figure 10, SGPT values for all participants are shown using violin and box-and-whisker plots. The median SGPT values of participants with HAV, HEV, and both infections were 543.90 (IQR, 404.45-898.05) IU/L, 532.20 (IQR, 392.70-1722.35) IU/L, and 544.70 (IQR, 393.43-1077.30) IU/L, respectively. The Kruskal-Wallis test and post hoc analysis with the Bonferroni adjustment revealed statistically significant differences (p < 0.001). Tables 5-7 show SGPT values for participants with HAV, HEV, and both infections, respectively. The trend analyses of these participants showed statistically significant differences (p < 0.001).

**Figure 11:**
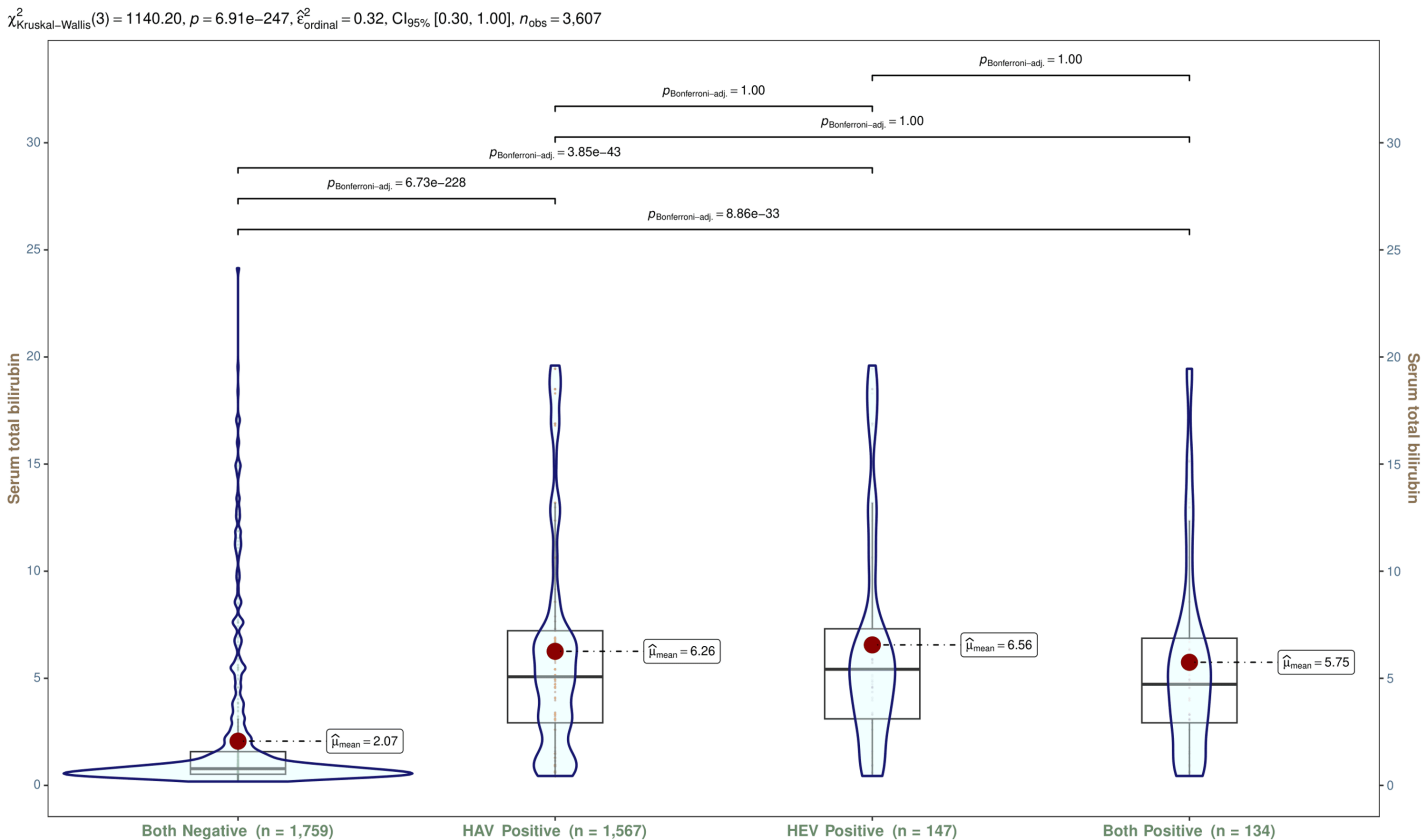
Serum total bilirubin values of all participants The box-and-whisker and violin plots show serum total bilirubin levels (mg/dL) for all participants (n = 3607). The normal range of serum total bilirubin is 0.1-1.2 mg/dL. The mean values are shown as red dots. The Kruskal-Wallis test was used to analyze these variables, and post hoc analysis was done with the Bonferroni adjustment. Statistical significance was set at p < 0.05. HAV: hepatitis A virus; HEV: hepatitis E virus

In Figure 11, the serum total bilirubin values for all participants are shown using violin and box-and-whisker plots. The median serum total bilirubin values of participants with HAV, HEV, and both infections were 5.07 (IQR, 2.92-7.22) mg/dL, 5.42 (IQR, 3.11-7.31) mg/dL, and 4.72 (IQR, 2.92-6.87) mg/dL, respectively. The Kruskal-Wallis test and post hoc analysis with the Bonferroni adjustment revealed statistically significant differences (p < 0.001). Tables 5-7 show serum total bilirubin values for participants with HAV, HEV, and both infections, respectively. The trend analyses of these participants showed statistically significant differences (p < 0.001).

## Discussion

This retrospective study presents a three-year overview of HAV and HEV seroprevalence among clinically suspected acute viral hepatitis cases at a tertiary care center. Participants with HAV infection outnumbered those with HEV infection or co-infection. Our findings concurred with those of Mahadevaiah et al. [10], but not with two other studies from India [2,15]. These data indicated regional differences in HAV and HEV infection. According to the Global Burden of Disease Study, there were approximately 160 million HAV infections worldwide in 2021, which resulted in 26,901 deaths. With roughly 30.6 million instances and 13,658 deaths, India was responsible for 19% of all HAV infections worldwide and nearly half of all HAV deaths [22]. Additionally, HEV infection or vaccination does not provide permanent protection as there could be reinfection with different HEV strains [23].

In our study, SGOT, SGPT, and serum total bilirubin levels were elevated among participants infected with HAV, HEV, or both. The proportion of participants from urban backgrounds increased each year. The cases with a history of any type of hepatitis decreased over time. These facts suggested either increased cases due to poor hygiene or increased awareness among urban residents. The proportions of each symptom remained similar across the years. The most common symptom in the study population (clinically suspected acute viral hepatitis) was abdominal pain, followed by fever, jaundice, and loose motion. The order held good for all three years. However, jaundice was the most common symptom among participants with one or both infections. The values of SGOT, SGPT, and serum total bilirubin were higher among HAV- and HEV-infected individuals than those of the non-infected ones. HAV and HEV infections damage hepatocytes, causing aberrant liver functioning. Jaundice is one of the symptoms of viral hepatitis. The liver enzymes SGOT and SGPT are crucial for protein processing. Inflammation or liver damage can result in elevated SGOT and SGPT levels [23,24].

HAV infection can only be prevented through vaccination and hygiene awareness. Like HAV, we have few pharmacotherapy options for the management of HEV infection. Owing to spontaneous HEV clearance, acute HEV infections are generally self-limiting. Reinfections with HEV can be prevented through increased awareness of hygiene practices and the development of vaccines [25].

Strengths and limitations

There are several advantages to this study, including a large sample size and subgroup analyses by year of hospital visit and infection type. Nevertheless, this study has some limitations. First, the retrospective design restricted us from clinical correlation of the laboratory data. Moreover, only clinically suspected cases that had been referred to serology laboratory testing were included in the study. These factors might lead to inaccurate results. Second, molecular typing could not be performed. Third, the clinical outcomes, incidence of acute liver failure, treatment course, and mortality rates could not be determined.

## Conclusions

HAV and HEV continue to play an important role in causing acute viral hepatitis. Age-specific distribution, seasonal patterns, and regional differences in the prevalence of HAV and HEV infections should be considered to prevent and manage such cases. HAV and HEV infections are public health issues in developing countries like India, owing to their large populations, high population density, inadequate hand hygiene practices, and a shortage of perennial water supply. Therefore, early screening, diagnosis, and treatment of suspected HAV and HEV infections could mitigate hospital burden and reduce morbidity and mortality. We recommend prospective studies to correlate antibodies with clinical outcomes and to assess their molecular typing.
